# *Clostridioides difficile* Toxin A-Induced Wnt/β-Catenin Pathway Inhibition Is Mediated by Rac1 Glucosylation

**DOI:** 10.3389/fmicb.2020.01998

**Published:** 2020-08-28

**Authors:** Conceição S. Martins, Deiziane V. S. Costa, Bruno B. Lima, Renata F. C. Leitäo, Gildênio E. Freire, Guilherme F. M. Silva, Dvison M. Pacífico, José G. Abreu, Gerly A. C. Brito

**Affiliations:** ^1^Postgraduate Program in Morphofunctional Sciences, Department of Morphology, School of Medicine, Federal University of Ceará, Fortaleza, Brazil; ^2^Department of Physiology and Pharmacology, School of Medicine, Federal University of Ceará, Fortaleza, Brazil; ^3^Department of Medicine, Division of Cardiology, Emory University School of Medicine, Atlanta, GA, United States; ^4^Department of Medical Sciences, School of Medicine, Federal University of Ceará, Fortaleza, Brazil; ^5^Department of Anatomy, Institute of Biomedical Sciences, Federal University of Rio de Janeiro, Rio de Janeiro, Brazil

**Keywords:** *Clostridioides difficile*, *Clostridioides difficile* toxin A, Rac1, Wnt, β-catenin

## Abstract

*Clostridioides difficile* toxin A (TcdA) has been shown to inhibit cellular Wnt signaling, the major driving force behind the proliferation of epithelial cells in colonic crypts, likely through the inhibition of β-catenin nuclear translocation. Herein, we aimed to advance the understanding of this mechanism by replicating the findings *in vivo* and by investigating the specific role of Rac1, a member of the Rho GTPase family, on the inhibition of the Wnt-induced β-catenin nuclear translocation triggered by TcdA. To investigate the effects of TcdA on the Wnt/β-catenin pathway *in vivo*, we injected the ileal loops of C57BL/6 mice with TcdA [phosphate-buffered saline (PBS) as the control] to induce *C. difficile* disease-like ileitis. After 4 h post-injection, we obtained ileum tissue samples to assess Wnt signaling activation and cell proliferation through Western blotting, immunohistochemistry, and qPCR. To assess the role of Rac1 on Wnt signaling inhibition by TcdA, we transfected rat intestinal epithelial cells (IEC-6) with either a constitutively active Rac1 plasmid (pcDNA3-EGFP-Rac1-Q61L) or an empty vector, which served as the control. We incubated these cells with Wnt3a-conditioned medium (Wnt3a-CM) to induce Wnt/β-catenin pathway activation, and then challenged the cells with TcdA. We assessed Wnt signaling activation *in vitro* with TOP/FOPflash luciferase assays, determined nuclear β-catenin translocation by immunofluorescence, measured cyclin D1 protein expression by Western blotting, and quantified cell proliferation by Ki67 immunostaining. *In vivo*, TcdA decreased β-catenin, cyclin D1, and cMYC expression and inhibited the translocation of β-catenin into the nucleus in the ileum epithelial cells. In addition, TcdA suppressed cell proliferation and increased Wnt3a expression, but did not alter Rac1 gene expression in the ileum tissue. *In vitro*, constitutively active Rac1 prevented Wnt signaling inhibition by enabling the β-catenin nuclear translocation that had been blocked by TcdA. Our results show that TcdA inhibits Wnt/β-catenin pathway *in vivo* and demonstrate that this inhibition is likely caused by a Rac1-mediated mechanism.

## Introduction

*Clostridioides difficile* (*C. difficile*), a gram positive, spore forming anaerobic bacterium, is a major cause of nosocomial diarrhea (Zhu et al., 2018). The two major virulence factors responsible for *C. difficile* infection are *C. difficile* toxin A (TcdA) and *C. difficile* toxin B (TcdB). TcdA and TcdB enter cells through receptor-mediated endocytosis and inhibit the small Rho guanosine triphosphatases (GTPases), such as Rho, Rac1, and Cdc42 (Chen et al., 2019). The Rho family of small GTPases is a branch within a superfamily of Ras-related small GTPases. Twenty mammalian genes encoding Rho GTPases have been identified of which Rac1, Cdc42, and RhoA are the prototypes and therefore the best characterized (Jaffe and Hall, 2005). These signaling proteins regulate all the actin-dependent process like cell migration, phagocytosis, and cell contraction. They are also involved in various other signaling pathways, regulating gene expression, cell cycle, and apoptosis. Most Rho GTPases cycle between a GTP-bound active conformation and a GDP-bound inactive conformation (Wennerberg and Der, 2004), acting as molecular switches. Not surprisingly, this family of molecules plays central roles in maintenance of health, and their dysregulation often results in disease. Anomalous signaling of Rho GTPases is found in many human cancers and may be attributed to several mechanisms, such as overexpression of Rho GTPases with oncogenic activity or alterations of upstream regulators or downstream effectors (Vega and Ridley, 2008).

GTPases are known to be critically involved in the regulation of intestinal epithelial barrier functions, since inhibition of Rho GTPases results in actin cytoskeleton disruption, apoptosis, and impairment of intestinal cell proliferation (Welsh et al., 2001; Brito et al., 2002; Lica et al., 2011; D’Auria et al., 2012; Chen et al., 2019). Most of these cellular processes are also regulated by the activated Wnt/β-catenin signaling pathway (Gao et al., 2017; Steinhart and Angers, 2018). A previous study from our research group showed that TcdA inhibits the Wnt/β-catenin signaling pathway in intestinal epithelial cells (IECs; Lima et al., 2014); however, its mechanism remains unknown.

The Wnt/β-catenin signaling pathway is a well-conserved and complex signaling cascade that plays an important role in development, homeostasis, and disease (Gough, 2012; Tortelote et al., 2017). This signaling pathway dictates intestinal epithelial layer self-renewal through a complex cellular process that includes cell proliferation, cell differentiation, and apoptosis (Ring et al., 2014). Pathway activation depends on the secretion of Wnt family proteins, which in turn bind to frizzled (FZD) receptors and low-density receptor-related protein (LRP) 5/6 coreceptors on the cell membrane (Wu et al., 2009). In the absence of Wnt ligands, the level of intracellular β-catenin is regulated by a multiprotein cytoplasmic degradation complex that consists of Axin and its interacting partners: tumor suppressor adenomatous polyposis coli (APC), glycogen synthase kinase 3 beta (GSK3β), and casein kinase 1 (CK1; Ring et al., 2014). The destruction complex phosphorylates β-catenin, leading to subsequent proteasomal degradation. When Wnt ligands bind their FZD and LRP5/6 receptors, the destruction complex is disassembled, allowing for β-catenin accumulation in the cytoplasm and its translocation to the nucleus. Then, nuclear β-catenin binds to the transcription factors of the T cell factor/lymphocyte enhancer factor (TCF/LEF) family (Kimelman and Xu, 2006; Ring et al., 2014) and promotes the transcription of Wnt/β-catenin pathway target genes, including *cMYC*, *cyclin D1*, *survivin*, *bcl-2*, *Rac1*, *Rho*, and *Cdc42* (Daniels and Weis, 2005; Lima et al., 2014).

Previous studies demonstrated that both TcdA and TcdB inhibit the Wnt/β-catenin pathway. Whereas TcdB inhibits this pathway by binding to the frizzled-7 (FZD-7) receptor in the colonic epithelium (Tao et al., 2016; Chen et al., 2019). However, whether TcdA impairs Wnt/β-catenin signaling transduction *in vivo* and the mechanism underlying its inhibitory action remain unknown. Given that Rac1 plays an important role in β-catenin translocation to the nucleus, we hypothesized that its inactivation by TcdA is crucial for Wnt/β-catenin signaling inhibition. Here, we investigated the role of Rac1 in the TcdA-mediated inhibition of the Wnt/β-catenin signaling pathway in IECs *in vitro* and characterized the impact of TcdA on Wnt/β-catenin signaling in a mouse ileal loop model.

## Materials and Methods

### Animals

All experimental protocols were approved by the Federal University of Ceará Committee on the Ethical Treatment of Research Animals (CEUA. N. 2727150218) and were performed in accordance with the Guide for Care and Use of Laboratory Animals (National Institutes of Health, Bethesda, MD, United States). Male C57BL/6 mice (8 weeks old) were obtained from the vivarium of the Department of Physiology and Pharmacology of the Federal University of Ceará. The mice were maintained in a temperature-controlled environment (22 ± 1°C) with a 12-h light/dark cycle and had a free access to drinking water and standard diet.

### *Clostridioides difficile* Toxin A-Induced Ileitis

TcdA-induced murine ileitis was induced as previously described (De Araújo Junqueira et al., 2011) with some modifications. Mice (*n* = 6 in each group) were fasted for 4 h and had a free access to water before being anesthetized (80 mg/kg ketamine and 10 mg/kg xylazine administered intraperitoneally). After midline laparotomy, a 4-cm ileal loop was ligated, and 10 μg of TcdA (TechLab) in 200 μl of phosphate-buffered saline (PBS) was injected at the site of the ligations. Control loops were injected with only 200 μl of PBS. After 4 h, the mice were euthanized (240 mg/kg ketamine and 15 mg/kg xylazine administered intraperitoneally), and the ileal loops were removed for subsequent analysis of the parameters defined in this study.

### Quantitative Real-Time PCR

Quantitative real-time PCR (qPCR) analysis of the gene expression of β-catenin, cyclin D1, cMYC, Wnt3a, and Rac1 was performed in mouse ileum tissue stored in RNAlater solution (Qiagen), an RNA stabilizer, at −80°C. Total RNA was extracted by an RNA isolation system (Promega) according to the manufacturer’s protocol. The RNA was quantified by a NanoDrop spectrometer (Thermo Fisher Scientific), and RNA quality was determined by examining the 260/280 ratio > 1.8. A total of 1 μg RNA was then reverse transcribed using a high-capacity cDNA reverse transcription kit (Applied Biosystems) according to the manufacturer’s protocol. qPCR was performed using SYBR Green PCR Master Mix (Applied Biosystems), as described in the manufacturer’s instructions. The sequences of the primers are listed in Table 1. To compare gene expression under different conditions, the expression under each condition (normalized to GAPDH, the endogenous control) was quantified relative to the control condition. For β-catenin, cyclin D1, cMYC, Wnt3a, and Rac1, qPCR amplification was performed in a CFX Connect system (Bio-Rad) under the following conditions: 50°C for 2 min and 95°C for 10 min, followed by 40 cycles of 95°C for 15 s and 60°C for 60 s. The relative expression levels of the genes were calculated using the threshold cycle (2^−ΔΔCT^) method (Livak and Schmittgen, 2001).

**Table 1:** Primers used in qPCR.

Gene	Forward	Reverse
*β-catenin*	ACGCACCATGCAGGAATACA	CTTAAGATGGCCAGCAAGC
*Cyclin D1*	GCGTACCCTGACACCAATCT	AATCTCCTTCTGCACGCACT'
*cMYC*	AGCTGCTTCGCCTAGAATTG	'CCTATTCAGCACGCTTCTCC
*Wnt3a*	TTCTTACTTGAGGGCGCAGA	AAGGAACCCAGATCCCAAAT
*Rac1*	GACCAGCCGACTAGCTTTTG3	CAGCACACCCACAACTAGGA
*GAPDH*	AGAACATCATCCCTGCATCC	CACATTGGGGGTAGGAACAC

### Measurement of Nuclear β-Catenin by Western Blotting

To obtain the nuclear extract from the ileum tissue samples, a nuclear extract kit (Abcam) was used according to the manufacturer’s protocol. Protein concentrations were determined through bicinchoninic acid assay according to the protocol of the kit manufacturer (Thermo Fisher Scientific). Fifty micrograms of reduced protein in Laemmli sample buffer with β-mercaptoethanol (Bio-Rad) was run on an SDS-PAGE gel (10%) and transferred onto PVDF membrane for 2 h. Then, the membranes were blocked with 5% blotting-grade blocker (Bio-Rad) for 1 h at room temperature and incubated overnight with anti-β-catenin (Abcam, 1:200) and anti-B23 (Santa Cruz Biotechnology, 1:1000) at 4°C. Then, the membranes were incubated with secondary antibody conjugated with horseradish peroxidase (HRP; Invitrogen, 1:1000) for 2 h at room temperature. The fluorescence of a chemiluminescent signal system was detected by a ChemiDoc™ XRS+ system (Bio-Rad, Life Technologies), and the bands were quantified by densitometry using Image Lab 5.0 software (Bio-Rad).

### Immunohistochemistry Assay for β-Catenin, Cyclin D1, cMYC, Ki67, and Wnt3a

Sections (4 μm thick) were prepared from paraffin-embedded ileum tissue. Then, the samples were deparaffinized, dehydrated, immersed in retrieval solution (DAKO, pH 6.0 or pH 9.0) for 30 min in PT Link (DAKO), incubated with 3% (v/v) hydrogen peroxide to block endogenous peroxidase for 20 min at room temperature, and washed in PBS. The sections were then incubated with β-catenin (Abcam, 1:400), cyclin D1 (DAKO Flex), cMYC (Abcam, 1:200), Ki67 (Abcam, 1:200), and Wnt3a (Invitrogen, 1:800) antibodies diluted in diluent antibody solution (DAKO) for 1 h at room temperature. Next, the sections were washed with wash buffer and incubated with polymer (HRP DAKO) for 30 min. The slides were then washed and stained with chromogen 3,3′-diaminobenzidine peroxide (DAB), followed by counterstaining with Mayer’s hematoxylin, dehydrated in a graded alcohol series, cleared with xylene, and placed on a coverslip. The negative controls were processed simultaneously as described above, with the primary antibody being replaced by the diluent antibody. The images were captured by means of a light microscope coupled to a camera with an LAZ 3,5 acquisition system (LEICA DM1000, Germany). One hundred epithelial cells were counted from each field to quantify the percentage of cells showing positive immunostaining for each protein.

### Cell Culture, Transfection and Wnt3a-Conditioned Medium Production

The rat intestinal epithelial cell (IEC-6) line was obtained from the repository of cells in Rio de Janeiro. IEC-6 cells were cultured in Dulbecco’s Modified Eagle’s Medium (DMEM, Gibco) supplemented with 10% fetal bovine serum (FBS), 1% antibiotics (100 μg/ml penicillin and 100 μg/ml streptomycin, Gibco) and 0.1 U/ml bovine insulin (Sigma) at 37°C in a humidified incubator under 5% CO_2_ conditions for 21–30 passages. For all the experiments, the IEC-6 cells were released using 0.25% trypsin-EDTA for 5 min.

The IEC-6 cells were transfected with pcDNA3-EGFP-Rac1-Q61L [Addgene, the vector pcDNA3 contains the mutant Rac1 encoding cDNA (Rac1) in its active form and the green fluorescent protein (GFP); Subauste et al., 2000] or an empty vector with Lipofectamine 3000 reagent (Thermo Scientific), as described in the manufacturer’s instructions. After 18 h, the transfection medium was removed, and the cells were incubated with 50% Wnt3a-conditioned medium (Wnt3a-CM) and/or TcdA (TechLab, 50 ng/ml). Then, after 24 h of incubation, the cells were collected for evaluation.

Wnt3a-CM was obtained from L cells stably transfected with the Wnt3a plasmid cultured in DMEM supplemented with 10% FBS for 4 days. The medium was collected and filtered using a 0.22 μm filter, and fresh medium was added to the cells and cultured for 3 more days. After this period, the medium was collected and mixed with the previous medium at a 1:1 ratio. As a control, the CM was similarly generated with an untransfected parental cell line.

### TCF Reporter Assay

The IEC-6 cells were cotransfected with TOPflash (Millipore)/FOPflash (Millipore) and the pRL-TK vector (Promega) in combination with the pcDNA3-EGFP-Rac1-Q61 or an empty vector with Lipofectamine 3000 reagent (Thermo Scientific) according to the reagent manufacturer’s instructions. TOPflash is a TCF reporter plasmid containing two sets of three copies of wild-type TCF-binding sites and is driven by a minimal promoter of thymidine kinase inserted upstream of the luciferase reporter gene. FOPflash contains mutated TCF-binding sites driven by the same thymidine kinase promoter and upstream luciferase open reading frame as used in TOPflash. FOPflash is used as a negative control for TOPflash activity. The pRL-TK vector contains the *Renilla* gene under the control of the constitutively active herpes simplex virus thymidine kinase promoter and was used as a normalizer (MAJOR, 007; Kumar, 2008). After transfection, the medium was removed, and 50% Wnt3a-CM with or without TcdA was added to the cells and incubated for 24 h. Next, the cells were lysed, and luciferase and *Renilla* activities were measured by a dual-luciferase reporter system (Promega) with a microplate luminometer. This assay is as a gold standard to evaluate β-catenin translocation into the nucleus.

### Immunofluorescence Staining and Confocal Microscopy

Immunofluorescence assays were performed as previously described (Lima et al., 2014). Briefly, IEC-6 cells were fixed in 4% paraformaldehyde in PBS at pH 7.6, washed with PBS, and permeabilized with 0.1% Triton X-100 in PBS for 5 min. The samples were then blocked for 1 h with PBS containing 5% bovine serum albumin (BSA). The cells were incubated with an anti-β-catenin antibody (1:200, Abcam) overnight at 4°C, and a secondary antibody conjugated with Alexa Fluor 596 (1:1000) for 1 h at room temperature. Then, the cells were incubated with 4′,6-diamidino-2-phenylindole (DAPI, Thermo Scientific) for 5 min, washed with PBS, and mounted with Prolong Gold mounting medium (Thermo Scientific). Images were captured using a CoolSNAP-Pro digital camera (Media Cybernetics, Bethesda, MD, United States). The percentage of cells showing positive immunostaining for β-catenin in the nucleus or cytoplasm and the percentage of unstained cells were determined by counting 100 DAPI-stained nuclei.

### Measurement of IEC-6 Cell Proliferation

IEC-6 cell proliferation was evaluated by Ki67 immunocytochemistry, as previously described by Tinoco-Veras et al. (2017). Briefly, 3 × 10^5^ cells/well in a 24-well plate were fixed with 4% paraformaldehyde solution for 30 min, washed with PBS, and permeabilized with 0.1% Triton X-100 for 5 min. The samples were then blocked for 1 h with 5% BSA and incubated with anti-Ki67 antibody (1: 200, Abcam) for 1 h at room temperature. The samples were then incubated with the HRP polymer (DAKO) for 30 min at room temperature. Then, the cells were stained with DAB chromogen (DAKO) and counterstained with hematoxylin. The coverslips were placed on slides using Faramount (DAKO). The percentage of cells showing positive immunostaining for Ki67 was determined by counting 100 hematoxylin-stained nuclei.

### Western Blot Analysis

Lysate samples from the treated IEC-6 cells were harvested in RIPA buffer (Thermo Scientific) by the procedure described by the manufacturer. Protein concentrations were determined through bicinchoninic acid assay according to the protocol of the kit manufacturer (Thermo Fisher Scientific). Thirty micrograms of reduced protein in Laemmli sample buffer with β-mercaptoethanol (Bio-Rad) was run on an SDS-PAGE gel (10%) and transferred onto PVDF membrane for 2 h. Then, the membranes were blocked with 5% blotting-grade blocker (Bio-Rad) for 1 h at room temperature and incubated overnight with anti-cyclin D1 (Abcam, ab32572, 1:200), anti-Rac1 (BD Bioscience, 610651, 1:500), and anti-β-actin (Santa Cruz Biotechnology, 1:1000) at 4°C. Then, the membranes were incubated with secondary antibody conjugated with HRP (Invitrogen, 1:1000) for 2 h at room temperature. The fluorescence of a chemiluminescent signal system was detected by a ChemiDoc™ XRS+ system (Bio-Rad, Life Technologies), and the bands were quantified by densitometry using Image Lab 5.0 software (Bio-Rad).

### Statistical Analysis

All quantitative results are expressed as the means ± standard error of the mean (SEM). Statistical analysis of the data was performed using GraphPad Prism software, version 6.0. Student’s *t*-tests were used to compare two groups, and analyses of variance (ANOVAs) followed by Bonferroni’s multiple comparison test were used for comparisons of more than two groups. A value of *p* < 0.05 was considered significant.

## Results

### TcdA Inhibits Wnt/β-Catenin Signaling *in vivo*

**Figure 1:**
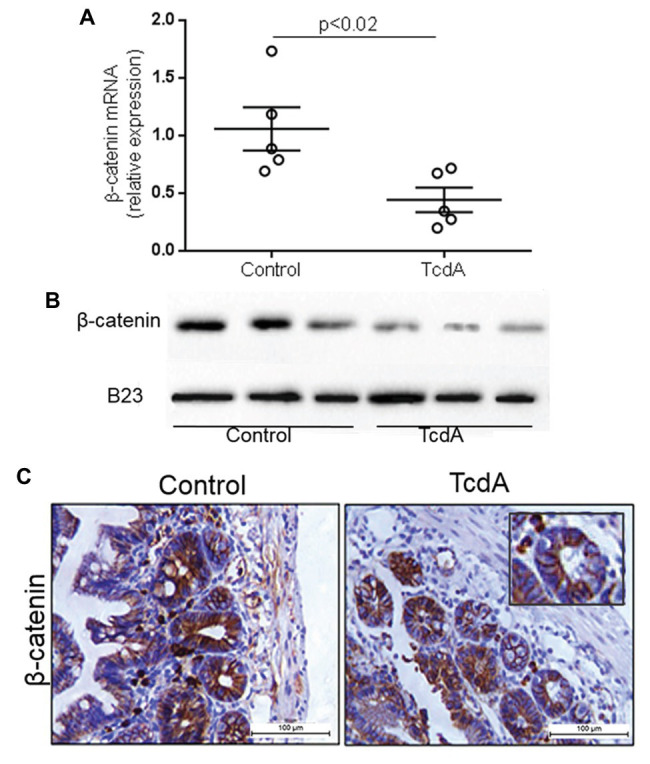
*Clostridioides difficile* toxin A (TcdA) decreases β-catenin expression and its nuclear translocation in mouse ileum. **(A)** *CTNNNB1* mRNA expression in the ileum of mice subjected to TcdA (10 μg)-induced ileitis as evaluated by qPCR. Bars represent the means ± SEM of five mice in each group; Student’s *t*-test. **(B)** The WB bands of each group showing β-catenin and B23 (a control protein) expression in the protein nuclear fraction in the ileum tissue. **(C)** Representative immunohistochemical images of β-catenin expression in mouse ileum control tissue and TcdA-treated ileal tissue.

Given that TcdA had previously been shown to inhibit Wnt/β-catenin signaling in the IEC-6 cells (Lima et al., 2014), we asked whether Wnt/β-catenin signaling could be inhibited in a mouse model of TcdA-induced ileitis, particularly since epithelial cells interact with other cells *in vivo*. We found that TcdA downregulated (*p* < 0.02) *β-catenin* gene expression in ileum tissue compared to the level in the control (Figure 1A). Similarly, β-catenin protein expression in the TcdA-challenged ileum tissue was notably reduced compared to that in control ileal tissue (Figure 1B). TcdA reduced the levels of β-catenin protein in the epithelial cell nuclei from ileal crypts (Figure 1C).

**Figure 2:**
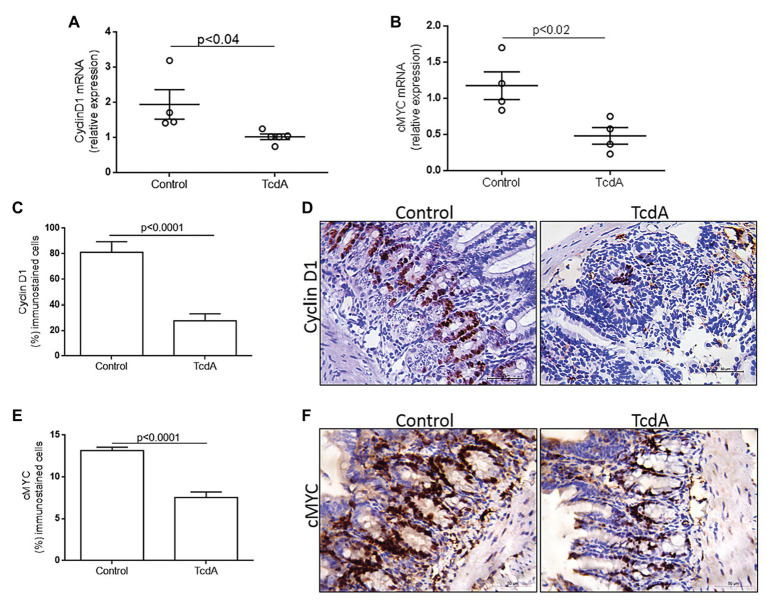
TcdA downregulates β-catenin target genes (*cMYC* and *cyclin D1*) and reduces cyclin D1 protein in the ileum of the treated mice. **(A)** *CCND1* and **(B)** *cMYC* mRNA expression in the ileum of mice treated with phosphate-buffered saline (PBS; control) or TcdA (10 μg) for 4 h, as evaluated by qPCR. Bars represent the means ± SEM of five mice in each group; Student’s *t*-test. **(C)** Percentage of the cells showing positive cyclin D1 immunostaining. Data are the means ± SEM; Student’s *t*-test. **(D)** Representative immunohistochemical images of cyclin D1 levels in the ilea of mice challenged with PBS (control) or TcdA (10 μg). **(E)** Percentage of the cells showing positive cMYC immunostaining. Data are the means ± SEM; Student’s *t*-test. **(F)** Representative immunohistochemical images of cMYC expression in mouse ilea challenged with PBS (control) or TcdA (10 μg).

Nuclear translocation of β-catenin activates TCF4, leading to the transcription of target genes, such as *cMYC* and *cyclin D1* (Chen et al., 2019). To analyze whether TcdA-induced inhibition of β-catenin nuclear translocation alters cMYC and cyclin D1 expression in the ilea of the mice, we performed a qPCR analysis of these genes. Notably, TcdA decreased *cMYC* (*p* < 0.02) and *cyclin D1* (*p* < 0.04) gene expression in the ilea of the treated mice compared to that in the ilea of the control group mice (Figures 2A,B). As observed in Figure 2C, TcdA reduced (*p* < 0.0001) cyclin D1 immunostaining in the ilea of the treated mice compared to that in the ilea of the control group mice. In the mouse ileum, cyclin D1 is mainly expressed by epithelial cells from ileal crypts (Figure 2D). TcdA also decreased (*p* < 0.0001) the number of cells showing cMYC immunostaining in the ilea of the treated mice compared to the number in the ilea of the control group mice (Figure 2E). As cyclin D1, cMYC is predominantly expressed by cells in ileal crypts in normal tissues, and its expression is reduced by challenge with TcdA (Figure 2F). These results are consistent with those showing Wnt/β-catenin target gene inhibition by TcdA and may indicate TcdA influence on cell proliferation.

### TcdA Decreases Cell Proliferation in Mouse Ileal Crypts

**Figure 3:**
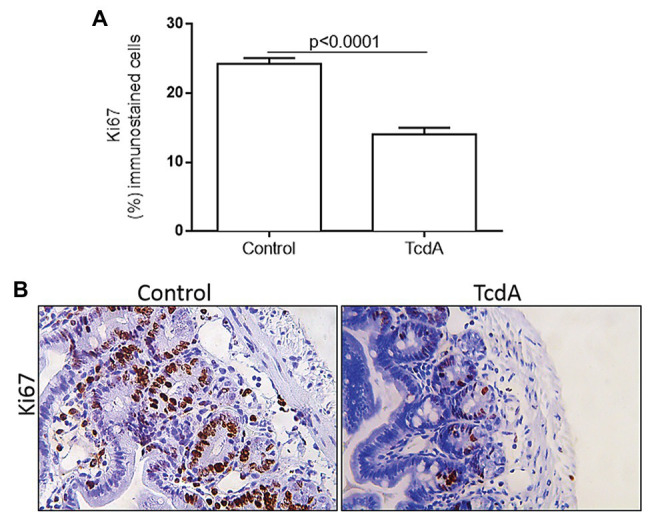
TcdA decreases cell proliferation in mouse ileum crypts. **(A)** Percentage of the cells showing positive Ki67 immunostaining. Data are the means ± SEM in 6–8 microscope fields per sample (*n* = 5 mice per group); Student’s *t*-test. **(B)** Representative immunohistochemical images of Ki67 levels in mouse ileum challenged with PBS (control) or TcdA (10 μg) for 4 h.

Because cMYC and cyclin D1 are proteins related to the cell proliferation cycle, we used Ki67 immunohistochemistry to evaluate whether TcdA is able to alter cell proliferation in the ileal crypts. We found that TcdA decreased the percentage of Ki67-positive cells in the ileal crypts of the treated mice compared with the percentage in the crypts of the control mice (*p* < 0.0001; Figure 3A). In the control mice, a greater number of Ki67-positive cells were found in intestinal crypts (Figure 3B), and they were considerably reduced by TcdA (Figure 3B).

### TcdA Upregulates Wnt3a in the Mouse Ileum

**Figure 4:**
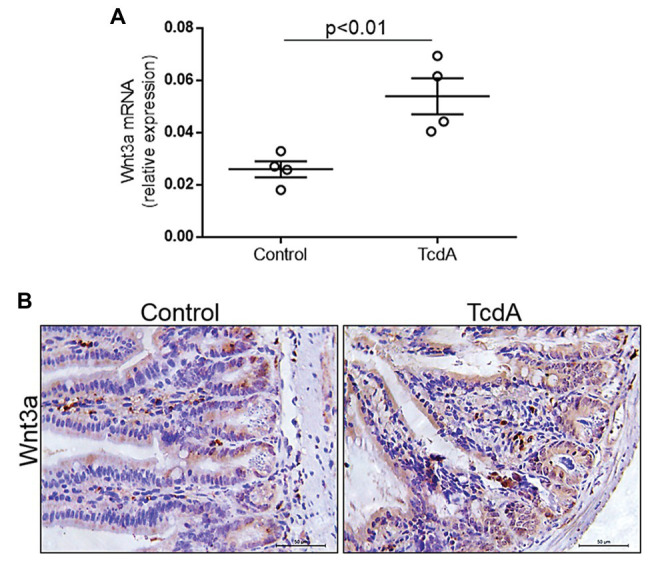
TcdA increases Wnt3a expression in the mouse ileum. **(A)** *Wnt3a* mRNA expression in the ilea of the mice treated with PBS (control) or TcdA (10 μg) for 4 h as evaluated by qPCR. Bars represent the means ± SEM of five mice in each group. Student’s *t*-test. **(B)** Immunohistochemical images of Wnt3a expression in the ilea of mice treated with PBS (control) or TcdA (10 μg) for 4 h.

Given that Wnt3a is an endogenous agonist that stimulates β-catenin translocation resulting in proliferation, we asked whether TcdA could affect its expression in the mouse ileum. We found that TcdA upregulated Wnt3a gene expression in the ilea of the treated mice compared to that in the ilea of the control group mice (*p* < 0.01; Figure 4A). TcdA increased Wnt3a protein expression in the epithelial cells (Figure 4B).

### TcdA Inhibition of β-Catenin Nuclear Translocation Induced by Wnt3a Is Reversed by Upregulation of Rac1 in the Epithelial Cells *in vitro*

**Figure 5:**
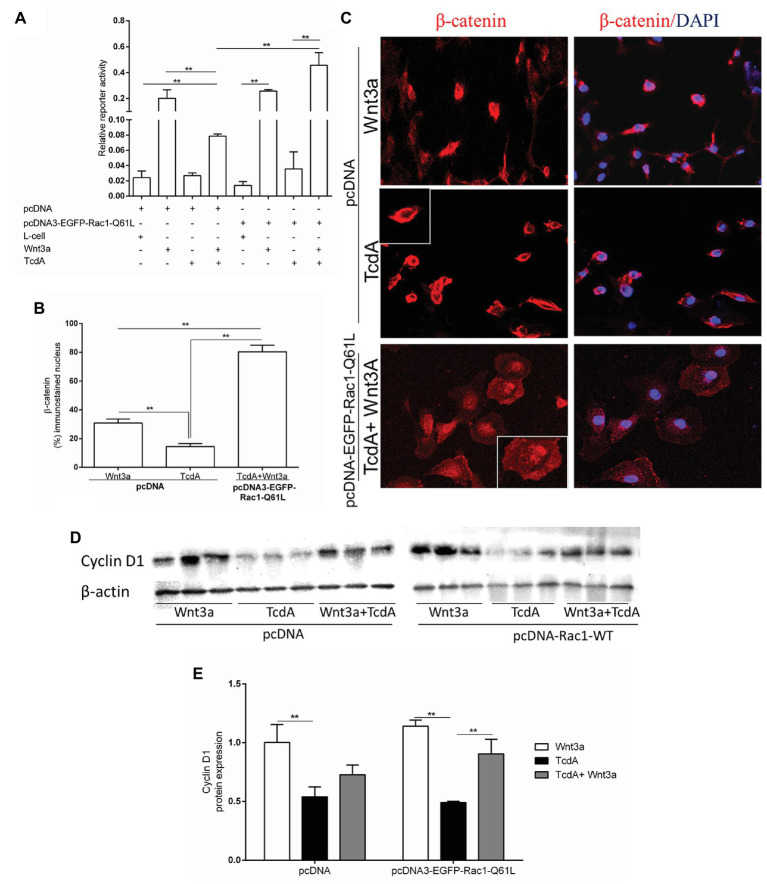
Upregulation of Rac1 reverses the TcdA inhibitory effect on β-catenin nuclear translocation induced by Wnt3a in intestinal epithelial cells (IEC-6). **(A)** Relative reporter activity of β-catenin/T cell factor (TCF) signaling in the IEC-6 cells cotransfected with pcDNA (empty vector) or pcDNA3-EGFP-Rac1-Q61L, and TOPflash luciferase reporter constructs followed by incubation with TcdA (50 ng/ml), Wnt3a-conditioned or L-cell medium for 24 h. *Renilla* luciferase constructs were used as an internal control for transfection efficiency. Bars represent the means ± SEM (*n* = 5). One-way ANOVA, followed by Bonferroni’s test, was used; ^**^p < 0.001. **(B)** Percentage of cells showing positive β-catenin immunostaining in the nucleus. Data are the means ± SEM. One-way ANOVA, followed by Bonferroni’s test, was used; ^**^p < 0.001. **(C)** Representative photomicrographs of immunostained β-catenin (red) and DAPI, a nuclear dye (blue), in IEC-6 cells transfected with pcDNA (empty vector) or pcDNA3-EGFP-Rac1-Q61L followed by 24 h incubation with TcdA (50 ng/ml) alone or Wnt3a-conditioned medium (Wnt3a-CM). **(D)** The WB bands of each group showing cyclin D1 and β-actin (a control protein) protein expression from lysed IEC-6 cells after 24 h of incubation. **(E)** Analysis of the relative band densities of cyclin D1 normalized to β-actin. Bars represent the means ± SEM (*n* = 5). One-way ANOVA, followed by Bonferroni’s test, was used; ^**^p < 0.001.

Previously, it was demonstrated that Wnt3a does not affect TcdA-induced β-catenin nuclear translocation inhibition (Lima et al., 2014). Because TcdA glucosylates Rho GTPases, such as Rac1 (without affecting Rac1 gene expression *in vivo*, as shown in Supplementary Figure 1), which induces the recruitment of β-catenin to the nucleus (Wu et al., 2008; Pethe et al., 2011; Jamieson et al., 2015), we investigated whether upregulation of Rac1 through transfection of pcDNA3-EGFP-Rac1-Q61L (as shown in Supplementary Figures 2, 3) could recover Wnt3a-induced β-catenin nuclear translocation in the intestinal epithelial (IEC-6) cells challenged with TcdA. We found that Wnt3a alone induced β-catenin nuclear activity in a manner independent of pcDNA3-EGFP-Rac1-Q61L transfection in the IEC-6 cells, while its transcriptional regulatory response (as demonstrated by the TOPflash/FOPflash, ratio) was inhibited by TcdA with either Wnt3a or Rac1 upregulation alone. Rac1 upregulation in the presence of Wnt3a-CM increased β-catenin nuclear activity in the IEC-6 cells challenged with TcdA (*p* < 0.001; Figure 5A). As observed in Figures 5B,C, in the IEC-6 cells transfected with pcDNA3-EGFP-Rac1-Q61L, Wnt3a alone increased the level of β-catenin immunostaining in the nucleus, and TcdA decreased the nuclear immunostaining in this epithelial cell line. Notably, Rac1 upregulation in the presence of Wnt3a-CM induced increased the level of β-catenin immunostaining in the nuclei of the IEC-6 cells challenged with TcdA (Figures 5B,C). As expected, the increased expression of Rac1 caused by transfection with pcDNA3-EGFP-Rac1-Q61L reversed the inhibitory effect of TcdA on cyclin D1 protein expression in the IEC-6 cells induced by Wnt3a compared with the expression in the IEC-6 cells transfected with pcDNA (empty vector) and incubated with TcdA and Wnt3a (Figures 5D,E).

### TcdA-Induced Inhibition of IEC-6 Cell Proliferation Is Recovered by the Upregulation of Rac1

**Figure 6:**
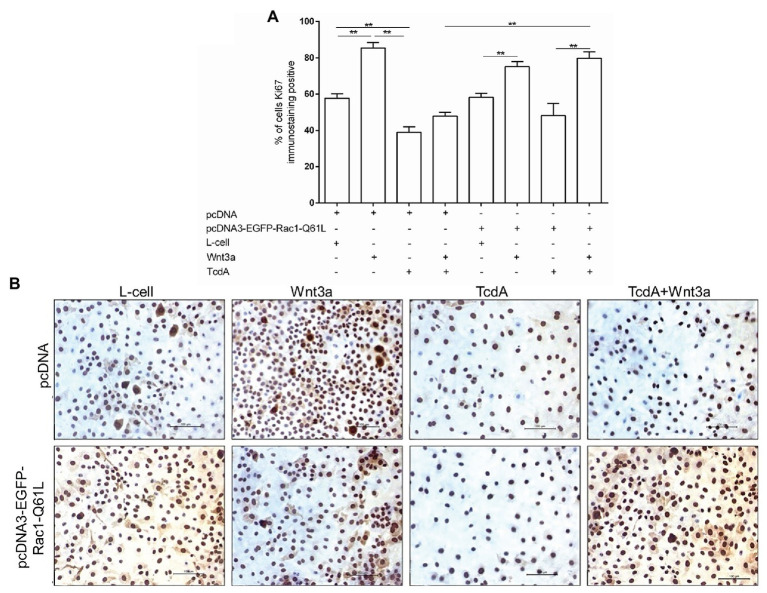
Upregulation of Rac1 reverses the TcdA inhibitory effect on Wnt3a-induced proliferation of the IEC-6 cells. **(A)** Percentage of IEC-6 cells showing positive Ki67 immunostaining after being cotransfected with pcDNA (empty vector) or pcDNA3-EGFP-Rac1-Q61L upon incubation with TcdA (50 ng/ml), Wnt3a-CM or L-cell medium for 24 h. Data are the means ± SEM (*n* = 6). One-way ANOVA, followed by the Bonferroni’s test, was used; ^**^p < 0.001. **(B)** Representative immunohistochemical images of the Ki67-immunostained IEC-6 cells transfected with pcDNA (empty vector) or pcDNA3-EGFP-Rac1-Q61L, followed by 24 h incubation with TcdA alone or Wnt3a-CM or L-cell medium.

Given that activation of the Wnt/β-catenin pathway results in cell proliferation and that TcdA inhibits the proliferation of IECs, we asked whether upregulation of Rac1 through the transfection of pcDNA3-EGFP-Rac1-Q61L could recover the proliferation of epithelial (IEC-6) cells challenged with TcdA, which we evaluated by immunostaining Ki67, which is a marker of cell proliferation. As shown in Figures 6A,B, neither Rac1 upregulation nor exposure to Wnt3a-CM affected the TcdA-induced inhibition of IEC-6 cell proliferation. However, the combination of Rac1 upregulation and Wnt3a-CM reversed the inhibitory effect of TcdA on IEC-6 cell proliferation (*p* < 0.001; Figures 6A,B).

## Discussion

Herein, we demonstrate that TcdA inhibits Wnt signaling *in vivo* by decreasing the translocation of β-catenin to the nucleus, and thus downregulating its target genes (*c-MYC* and *cyclin D1*). Through this pathway, TcdA was shown to inhibit intestinal crypt cell proliferation in a mouse model of *C. difficile*-induced disease-like ileitis despite Wnt3a upregulation observed in the crypts and lamina propria cells, which were insufficient to induce Wnt/β-catenin activation.

Our laboratory had previously demonstrated that TcdA inhibits the Wnt/β-catenin pathway in IECs *in vitro* even when the cells are in the presence of the activator Wnt3a, an endogenous agonist; lithium chloride, a GSK3β inhibitor; and/or z-VAD-fmk, a nonspecific caspase inhibitor (Lima et al., 2014). Here, we demonstrated that Rac1 upregulation in the presence of Wnt3a-CM rescued TcdA-induced transcriptional inhibition (as demonstrated by the TOPflash/*Renilla* ratio), β-catenin nuclear translocation, cyclin D1 protein expression, and cell proliferation, implying that TcdA inhibits the Wnt/β-catenin pathway in a Rac1-dependent manner.

As shown previously, the inhibitory effects of TcdA on the Wnt/β-catenin pathway persist even in the presence of constitutively active β-catenin (Lima et al., 2014), suggesting that its inhibitory effect may be related to a defect in the process of β-catenin translocation to the nucleus, a process in which Rac-1 has been shown to play a crucial role (Buongiorno et al., 2008; Wu et al., 2008, 2009; Jamieson et al., 2015). Rac1 activates JNK, which in turn phosphorylates β-catenin, promoting its translocation to the nucleus (Wu et al., 2008).

Although Rac1 mRNA expression is not modified by TcdA, as shown in Supplementary Figure 1, it is well-known that TcdA, as well as TcdB, glucosylates Rho GTPases, such as, RhoA/B/C, Rac1/2, and Cdc42 promote Rac1 inactivation, decreasing the level of the active Rac1 isoform in the cytoplasm, and thus impeding its biological function (Reineke et al., 2007; Gerhard et al., 2008; Genisyuerek et al., 2011; Chen et al., 2016).

Using a promoter of the active pcDNA3-EGFP-Rac1-Q61L plasmid to upregulate Rac1 in IEC-6 cells (as shown in Supplementary Figure 3), we found that an increase in constitutively active Rac1 reverses both the inhibition of β-catenin translocation to the nucleus and the decrease in IEC-6 cell proliferation induced by TcdA in an extracellular Wnt3a-dependent manner. These data suggest that inhibition of the Wnt/β-catenin signaling pathway promoted by TcdA is a consequence of Rac1 glucosylation and inactivation by this toxin. The effect of EHop-016, a Rac inhibitor, on cell proliferation in mammary tumor growth reinforces our hypothesis (Montalvo-Ortiz et al., 2012). In agreement with our data, a previous report demonstrated that a dominant-negative Rac1 mutant drastically inhibits Wnt signaling in colon cancer followed by a decrease in its target gene transcription (Esufali and Bapat, 2004). Moreover, in *Drosophila* embryos, the deletion of *RacGAP50C*, a gene that negatively regulates Rac1, promoted the activation of Wnt/β-catenin (Jones and Bejsovec, 2005). Rac1 also plays an important role in the phosphorylation of LRP5/6, a coreceptor required for FZD activation by Wnt proteins, in a PIP2-dependent manner (Schlessinger et al., 2009). PIP2 synthesis requires PIP4K and PIP5K, which are activated by Rho and Rac1 (Schlessinger et al., 2009). It is possible that inactivation of Rac1 by TcdA affects different stages of Wnt/β-catenin signaling, but further investigations are needed to determine the exact stages affected.

TcdA and TcdB can act in different Rho proteins, but previous study suggested that Rac1, rather than RhoA or Cdc42, is crucial for the cytopathic effects induced by TcdA and TcdB (Halabi-Cabezon et al., 2008). Although, we may not exclude the role of other Rho proteins, rather than Rac1, on TcdA inhibitory effect on Wnt/β-catenin signaling pathway, the reversion of this effect by Rac1 upregulation suggests that Rac1 is a main mediator in this inhibition. In addition, we cannot exclude the involvement of additional mechanisms to modulate the activity of Rac1 in the present study, considering that specific modification at a single threonine residue in the small GTPases leads these important key players of several signaling pathways to their functional inactivation. TcdA and TcdB glucosylate Rac1/Cdc42 at threonine-35 and RhoA, decreasing their active levels into the cells (Halabi-Cabezon et al., 2008; Schoentaube et al., 2009; Genth et al., 2018).

Rac1 involvement in TcdB-induced cytopathic effects in epithelial cells has been demonstrated (Halabi-Cabezon et al., 2008). In turn, inactivation of Rac1 is involved in the cytotoxic effect of high concentrations of TcdB (Beer et al., 2018). However, in our investigation, the upregulation of constitutively active Rac1 alone, without a Wnt3a conditioned medium, did not rescue the antiproliferative effect of TcdA or the inhibition of β-catenin translocation, suggesting that these toxins may act by different mechanisms. In addition, whereas TcdB has been shown to inhibit the activity of the Wnt/β-catenin signaling pathway by binding to the FZD-7 receptor expressed by cells in the colonic epithelium (Tao et al., 2016), thereby preventing its activation by Wnt3a, TcdA has not shown affinity for FZD2, FZD3, or FZD-7 (Gupta et al., 2017). Like TcdA, TcdB induces inactivation of Rho GTPases including Rac-1 (Halabi-Cabezon et al., 2008; Schoentaube et al., 2009; Genth et al., 2018), which should cause Wnt/β-catenin signaling pathway inhibition. However, the investigation on TcdB effect in this pathway was not the aim of this study.

Wnt/β-catenin pathway activation with subsequent recruitment of β-catenin into the nucleus results in epithelial cell proliferation due to the increased expression of key cell cycle proteins, such as cyclin D1 and c-MYC (Clevers, 2006; Humphries and Wright, 2008). These protein expression levels were reduced by TcdA followed by a decrease in epithelial proliferation in the mouse ileum, as demonstrated in the present study. Consistently, TcdA critically affected three of the most important aspects of the intestinal mucosal repair process: epithelial cell migration, apoptosis, and cell proliferation (Brito et al., 2005). In addition, another report demonstrated that β-catenin‐ and TCF4-knockdown mice exhibited decreased cell proliferation in intestinal crypts (Tao et al., 2014). Multiple pathways, including the Wnt/β-catenin pathway and the expression of their target genes, are essential for maintaining epithelial barrier function and epithelial cell repair after injury. In *C. difficile* infection, the accumulation of TcdA and TcdB is associated with Wnt/β-catenin pathway inhibition, although this downregulation is induced, at least in part, by different mechanisms, and together with pro-inflammatory cytokine production within the intestinal mucosa, this accumulation likely results in intestinal epithelial stem cell niche degeneration and suppression of repair (Farin et al., 2014; Leslie et al., 2015; Liu et al., 2019).

Interestingly, the inhibition of β-catenin translocation to the nucleus and the subsequent downregulation of β-catenin target genes were maintained even during TcdA-induced Wnt3a upregulation in the mouse ileum, suggesting that a TcdA intracellular mechanism is involved in its inhibitory effect on the Wnt/β-catenin pathway. According to our data, increased Wnt3a expression was found in patients with ulcerative colitis compared to the level found in patients with noninflammatory bowel disease, but no difference was found in the expression of a panel of Wnt target genes (You et al., 2008). Although Paneth cells are an important source of Wnt ligands (Wnt3a) needed to sustain the self-renewal of intestinal epithelial stem cells, Paneth cells are impaired upon *C. difficile* infection (Liu et al., 2019). In the present study, the expression of Wnt3a was found mainly in lamina propria cells, suggesting that immune-induced inflammatory cells are involved in the release of Wnt3a. According to this premise, several studies have reported the synthesis of Wnt ligands by macrophages, which are related to mucosal healing (Loilome et al., 2014). There is a possibility that increased if Wnt3a upregulation may result in simultaneous upregulation of Wnt3a inhibitors, such as DKK and SFRP, which in turn additionally could contribute to pathway inhibition.

In addition to the importance of the clarification of this pathway inhibition mechanism by TcdA in *C. difficile* infection, targeting Rac-1 with TcdA, which resulted in the Wnt/β-catenin pathway and cell proliferation inhibition, may be one strategy to control tumor growth. According to this hypothesis, new findings suggest that TcdA may be able to inhibit proliferation and apoptosis and partially reverse multidrug resistance in a human chronic myeloid leukemia cell line (Xi et al., 2018).

**Figure 7:**
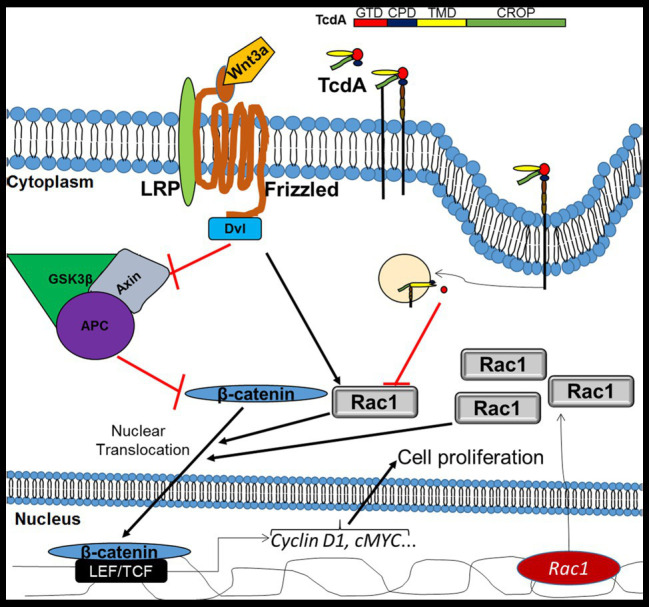
Mechanism of inhibition of Wnt/β-catenin signaling by TcdA. In IECs, Wnt3a binds to the frizzled (FZD) receptor and its coreceptor lymphoid enhancer factor (LRP), recruiting disheveled (Dvl), which in turn inhibits the adenomatous polyposis coli (APC)/glycogen synthase kinase 3 beta (GSK3β)/Axin complex, which degrade β-catenin. Upon inhibition of the APC/GSK3β/APC complex, β-catenin accumulates in the cytoplasm and is translocated into the nucleus, with Rac1 serving as a promoter of this translocation, where it activates LEF/TCF to promote the transcription of target genes, such as *cyclin D1* and *cMYC*. Both cyclin D1 and cMYC are involved in the induction of cell proliferation. TcdA [composed of glucosyltransferase domain (GDT), cysteine protease domain (CPD), translocation membrane domain (TMD), and C-terminal combined repetitive oligopeptide (CROP)] binds to a cell receptor by CROP and is endocytosed. Then, GDT is released into the cytosol, where it inhibits Rac1, resulting in a decrease in β-catenin translocation to the nucleus with a consequent reduction in the genic expression of the target genes and cell proliferation. However, upregulation of Rac1, in the presence of Wnt3a activity, decreases TcdA-induced inhibition of Wnt3a/β-catenin signaling and proliferation. APC, adenomatous polyposis coli; CPD, cysteine protease domain; CROPs, C-terminal combined repetitive oligopeptides; Dvl, disheveled; GSK3β, glycogen synthase kinase 3 beta; GTD, glucosyltransferase domain; LEF, lymphoid enhancer factor; LRP, low-density-lipoprotein-related protein; TCF, T cell factor protein; and TMD, translocation membrane domain.

Taken together, our data suggest that Rac1 inactivation is one of the mechanisms behind the impairment of Wnt/β-catenin signaling pathway by TcdA (Figure 7). The clarification of the involvement of Rac-1 in the mechanism of *C. difficile* TcdA inhibition reveals a potential target for future research on the modulation of Wnt/β-catenin signaling for therapeutic interventions.

## Data Availability Statement

All datasets generated for this study are included in the article/Supplementary Material.

## Ethics Statement

The animal study was reviewed and approved by Federal University of Ceará Committee on the Ethical Treatment of Research Animals (CEUA. No. 2727150218).

## Author Contributions

CM designed and performed all the experiments, analyzed the data, wrote the manuscript, and helped in the acquisition of the data. GS, GF, and DP performed experiments and contributed to the data analysis. DC, RL, BL, and JA helped with the experimental design and the revision of the manuscript. GB, the principal investigator for the grant and for constructing the experimental design, supervised the project and helped write the manuscript. All authors contributed to the article and approved the submitted version.

### Conflict of Interest

The authors declare that the research was conducted in the absence of any commercial or financial relationships that could be construed as a potential conflict of interest.
