# Supplementary material for: Clostridioides difficile Toxin A-Induced Wnt/β-Catenin Pathway Inhibition Is Mediated by Rac1 Glucosylation
Source: Front Microbiol. 2020 Aug 28;11:1998. doi: 10.3389/fmicb.2020.01998 (PMC7483921; doi:10.3389/fmicb.2020.01998)
Supplement: Supplementary file 1 [file Data_Sheet_1.doc]

*Clostridioides difficile* **Toxin A-Induced Wnt/β-catenin Pathway Inhibition Is Mediated by Rac1 Glucosylation**

Conceição S. Martins^1^, Deiziane V.S. Costa^2^, Bruno B. Lima^3^, Renata F. C. Leitäo^1^, Gildênio E. Freire¹, Guilherme F. M. Silva^4^; Dvison M. Pacífico^4^, José G. Abreu^5^, Gerly A. C Brito^1,2^*

^1^Postgraduate Program in Morphofunctional Sciences, Department of Morphology, School of Medicine, Federal University of Ceará, Fortaleza, Ceará, Brazil.

^2^Department of Physiology and Pharmacology, School of Medicine, Federal University of Ceará, Fortaleza, Ceará, Brazil.

^3^Department of Medicine, Division of Cardiology, Emory University School of Medicine, Atlanta, GA, USA.

^4^Department of Medical Sciences, School of Medicine, Federal University of Ceará, Fortaleza, Ceará, Brazil.

^5^Institute of Biomedical Sciences, Federal University of Federal University of Rio de Janeiro, Brazil.

Table 1 Primers used in qPCR

| Gene | Foward | Reverse |
| --- | --- | --- |
| *β-catenin* | ACGCACCATGCAGGAATACA | CTTAAGATGGCCAGCAAGC |
| *Cyclin D1* | GCGTACCCTGACACCAATCT | AATCTCCTTCTGCACGCACT’ |
| *CMYC* | AGCTGCTTCGCCTAGAATTG | ’CCTATTCAGCACGCTTCTCC |
| *Wnt3a* | TTCTTACTTGAGGGCGCAGA | AAGGAACCCAGATCCCAAAT |
| *Rac1* | GACCAGCCGACTAGCTTTTG3 | CAGCACACCCACAACTAGGA |
| *GAPDH* | AGAACATCATCCCTGCATCC | CACATTGGGGGTAGGAACAC |

**Supplementary figure 1**

*
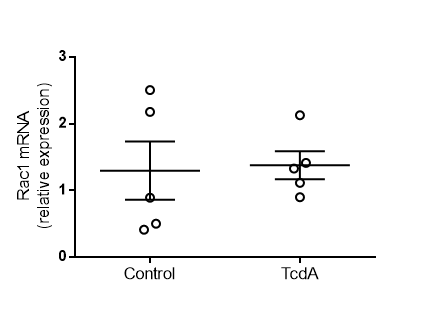
*

*Rac1* mRNA expression in the mouse ileum treated with PBS (control) or TcdA evaluated by qPCR. Bars represent the means ± SEM of 5 mice in each group; Student's t test.

**Supplementary figure 2**


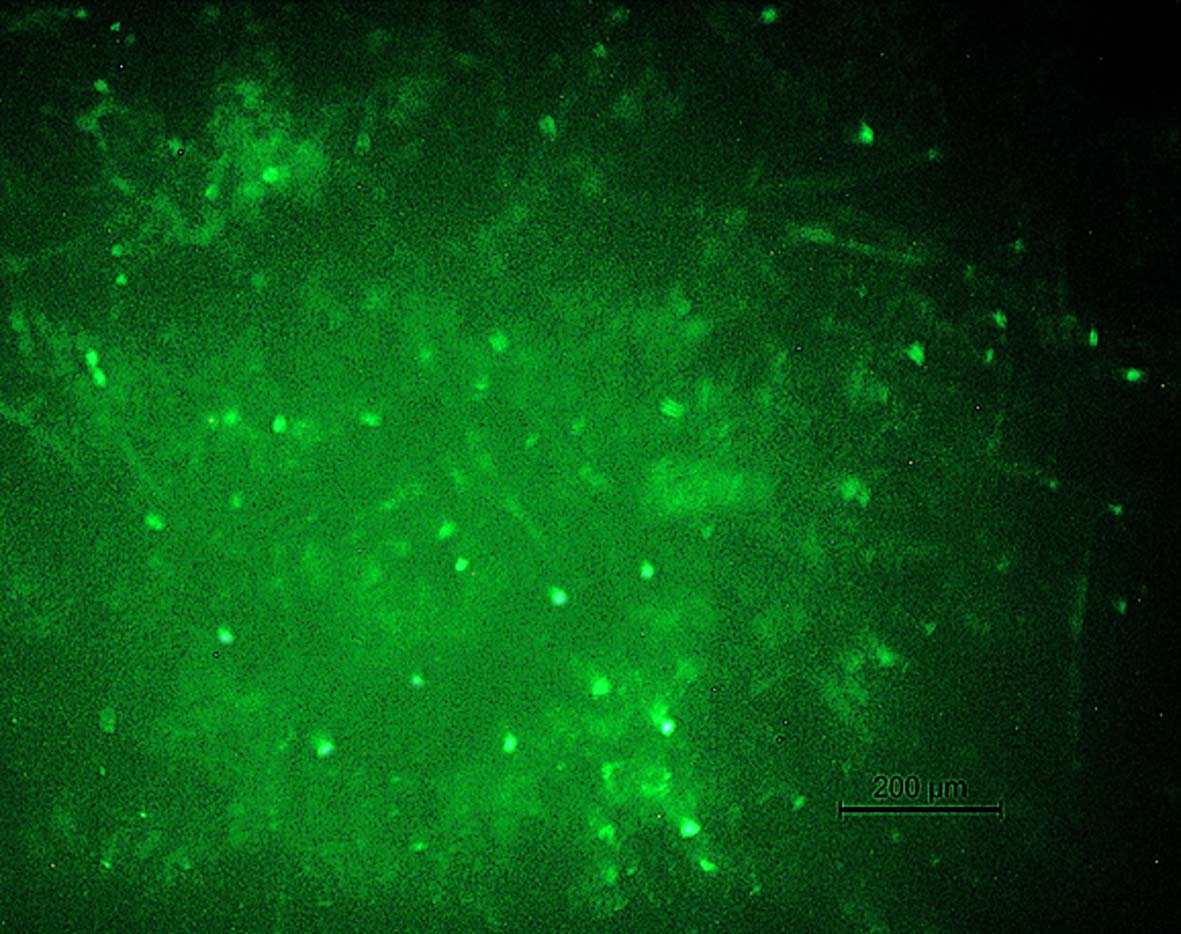


IEC-6 cells transfected with pcDNA3-EGFP-Rac1-Q61L after 18h. Cells containing the GFP plasmid (green) was obtained under the immunofluorescence microscope.

**Supplementary figure 3**

**
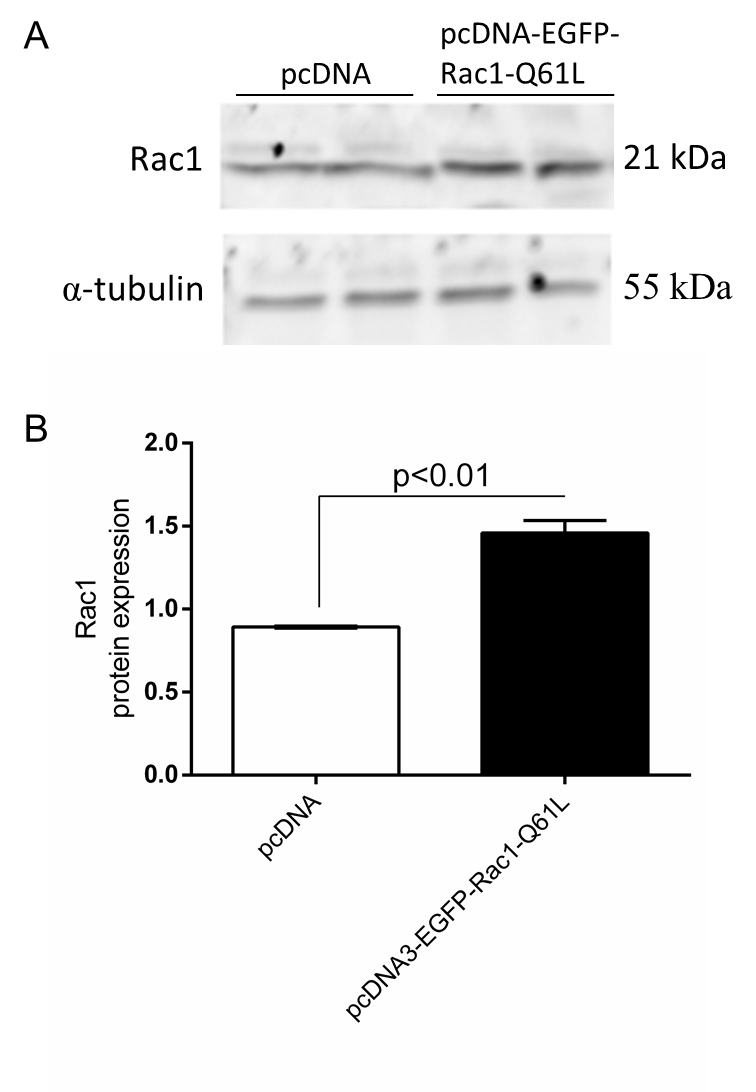
**

IEC-6 cells transfected with pcDNA3-EGFP-Rac1-Q61L or pcDNA (empty vector) after 24h. (A) The WB bands of each group showing Rac1 and α-tubulin (a control protein) protein expression from lysed IEC6 cells after 24 h of transfection. (E) Analysis of the relative band densities of Rac1 normalized to α-tubulin. Bars represent the means ± SEM (n=5). Student´s t test.
